# Automatically Explaining Machine Learning Prediction Results on Asthma Hospital Visits in Patients With Asthma: Secondary Analysis

**DOI:** 10.2196/21965

**Published:** 2020-12-31

**Authors:** Gang Luo, Michael D Johnson, Flory L Nkoy, Shan He, Bryan L Stone

**Affiliations:** 1 Department of Biomedical Informatics and Medical Education University of Washington Seattle, WA United States; 2 Department of Pediatrics University of Utah Salt Lake City, UT United States; 3 Care Transformation and Information Systems Intermountain Healthcare Salt Lake City, UT United States

**Keywords:** asthma, forecasting, machine learning, patient care management

## Abstract

**Background:**

Asthma is a major chronic disease that poses a heavy burden on health care. To facilitate the allocation of care management resources aimed at improving outcomes for high-risk patients with asthma, we recently built a machine learning model to predict asthma hospital visits in the subsequent year in patients with asthma. Our model is more accurate than previous models. However, like most machine learning models, it offers no explanation of its prediction results. This creates a barrier for use in care management, where interpretability is desired.

**Objective:**

This study aims to develop a method to automatically explain the prediction results of the model and recommend tailored interventions without lowering the performance measures of the model.

**Methods:**

Our data were imbalanced, with only a small portion of data instances linking to future asthma hospital visits. To handle imbalanced data, we extended our previous method of automatically offering rule-formed explanations for the prediction results of any machine learning model on tabular data without lowering the model’s performance measures. In a secondary analysis of the 334,564 data instances from Intermountain Healthcare between 2005 and 2018 used to form our model, we employed the extended method to automatically explain the prediction results of our model and recommend tailored interventions. The patient cohort consisted of all patients with asthma who received care at Intermountain Healthcare between 2005 and 2018, and resided in Utah or Idaho as recorded at the visit.

**Results:**

Our method explained the prediction results for 89.7% (391/436) of the patients with asthma who, per our model’s correct prediction, were likely to incur asthma hospital visits in the subsequent year.

**Conclusions:**

This study is the first to demonstrate the feasibility of automatically offering rule-formed explanations for the prediction results of any machine learning model on imbalanced tabular data without lowering the performance measures of the model. After further improvement, our asthma outcome prediction model coupled with the automatic explanation function could be used by clinicians to guide the allocation of limited asthma care management resources and the identification of appropriate interventions.

## Introduction

### Background

About 8.4% of Americans have asthma [1]. Each year in the United States, asthma costs over US $50 billion and results in more than 2 million emergency department (ED) visits, about half a million inpatient stays, and more than 3000 deaths [1,2]. A major goal in managing patients with asthma is to reduce their hospital visits, including ED visits and inpatient stays. As employed by health plans in 9 of 12 metropolitan communities [3] and by health care systems such as Intermountain Healthcare, Kaiser Permanente Northern California [4], and the University of Washington Medicine, the state-of-the-art method for achieving this goal is to employ a predictive model to predict which patients with asthma are highly likely to have poor outcomes in the future. Once identified, such patients are enrolled in care management. Care managers then call these patients on the phone regularly and help them make appointments for health and related services. By offering such tailored preventive care properly, up to 40% of future hospital visits by patients with asthma can be avoided [5-8].

A care management program has limited enrollment capacity [9]. As a result, the effectiveness of the program depends critically on the accuracy of the predictive model. Not enrolling a patient who is likely to have future hospital visits in the program is a missed opportunity to improve the patient’s outcomes. Unnecessarily enrolling a patient who is likely to have no future hospital visit would increase health care costs and waste scarce care management resources with no potential benefit. The current models for predicting hospital visits in patients with asthma are inaccurate, with published sensitivity of ≤49% and an area under the receiver operating characteristic curve (AUC) ≤0.81 [4,10-22]. When employed for care management, these models miss more than half of the patients who will have future hospital visits and erroneously label many other patients as likely to have future hospital visits [23]. To address these issues, we recently built an extreme gradient boosting (XGBoost) [24] machine learning model to predict asthma hospital visits in the subsequent year in patients with asthma [23]. Compared with previous models, our model raised the AUC by at least 0.049. However, like most machine learning models, our model offers no explanation of its prediction results. This creates a barrier for use in care management, where care managers need to understand why a patient is at risk for poor outcomes to make care management enrollment decisions and identify suitable interventions for the patient.

### Objectives

To overcome the abovementioned barrier, this study aims to develop a method to automatically explain the prediction results of our model and recommend tailored interventions without lowering any of the performance measures of our model, such as AUC, accuracy, sensitivity, specificity, positive predictive value, and negative predictive value.

In the following sections, we describe our methods and the evaluation results. A list of abbreviations adopted in this paper is provided at the end of the paper.

## Methods

We used the same patient cohort, data set, prediction target, cutoff threshold for binary classification, method for data preprocessing, including data cleaning and data normalization, and method for partitioning the whole data set into the training and test sets that we described in our prior paper [23].

### Ethics Approval and Study Design

This study consists of a secondary analysis of retrospective data and was evaluated and approved by the institutional review boards of the University of Washington Medicine, University of Utah, and Intermountain Healthcare.

### Patient Population

Our patient cohort included all patients with asthma who received care at any Intermountain Healthcare facility between 2005 and 2018 and resided in Utah or Idaho as recorded at the visit. Intermountain Healthcare is the largest health care system in Utah and southeastern Idaho. It operates 185 clinics and 22 hospitals and provides care for approximately 60% of people living in that region. A patient was considered asthmatic in a specific year if in the encounter billing database, the patient had one or more asthma diagnosis codes during that year (International Classification of Diseases, ninth revision [ICD-9]: 493.0x, 493.1x, 493.8x, 493.9x; International Classification of Diseases, tenth revision [ICD-10]: J45.x) [12,25,26]. The only exclusion criterion from the analysis in any given year was patient death during that year.

### Data Set

We used a structured clinical and administrative data set provided by the enterprise data warehouse of Intermountain Healthcare. The data set covered all visits by the patient cohort within Intermountain Healthcare between 2005 and 2018.

### Prediction Target (the Dependent or Outcome Variable)

For each patient identified as asthmatic in a specific year, the outcome was whether any asthma hospital visit occurred in the subsequent year. In this paper, an asthma hospital visit refers to an ED visit or an inpatient stay at an Intermountain Healthcare facility with a principal diagnosis of asthma (ICD-9: 493.0x, 493.1x, 493.8x, 493.9x; ICD-10: J45.x). For training and testing the XGBoost model and automatic explanation method, data of every patient with asthma up to the end of every year were used to predict the patient’s outcome in the subsequent year.

### Predictive Model and Features (Independent Variables)

Our recent XGBoost model [23] uses 142 features to predict asthma hospital visits in the subsequent year in patients with asthma. As listed in the multimedia appendix in our previous study [23], these features were computed from the structured attributes in our data set covering a wide range of categories, such as patient demographics, visits, medications, laboratory tests, vital signs, diagnoses, and procedures. Each input data instance for our model has these 142 features, targets a pair of a patient with asthma and a year, and is employed to predict the patient’s outcome in the subsequent year. We set the cutoff threshold for binary classification at the top 10% of patients with asthma having the largest predicted risk. These patients were predicted to incur asthma hospital visits in the subsequent year.

### Automatic Explanation Method

Previously, we developed an automated method to offer rule-formed explanations for any machine learning model’s prediction results on tabular data and recommend tailored interventions without lowering the performance measures of the model [27,28]. Our method was initially demonstrated to predict the diagnosis of type 2 diabetes [27]. Later, other researchers successfully applied our method to predict death or lung transplantation in patients with cystic fibrosis [29], predict cardiac death in patients with cancer, and use predictions to manage preventive care, heart transplant waiting list, and posttransplant follow-ups in patients with cardiovascular diseases [30]. In our method, each rule used for providing explanations has a performance measure termed confidence that must be greater than or equal to a given minimum confidence threshold *c_min_*. Our original automatic explanation method [27] was designed for reasonably balanced data, where distinct values of the outcome variable appear with relatively similar frequencies. Recently, we outlined an extension of this method [31,32] to handle imbalanced data, where one value of the outcome variable appears much less often than another. This data imbalance exists when predicting asthma hospital visits in patients with asthma, where only about 4% of the data instances are linked to future asthma hospital visits [23]. In our extended method, each rule used for providing explanations has a second performance measure termed commonality, which must be greater than or equal to a given minimum commonality threshold *m_min_*. To date, no technique has been developed to efficiently mine the rules with commonality greater than or equal to *m_min_*, compute their confidence, and eliminate those rules with confidence less than *c_min_* in the extended method, despite such techniques being essential for handling large data sets. No guideline exists for setting the values of the parameters used in the extended method, although they greatly impact the performance of the extended method. The extended method has never been implemented in computer code. Moreover, the effectiveness of the extended method has not been evaluated or demonstrated.

In this study, we made the following innovative contributions:

1. We provide several techniques for efficiently mining the rules with commonality greater than or equal to *m_min_*, computing their confidence, and eliminating those rules with confidence less than *c_min_* in the extended automatic explanation method. This completes our extended method. Although our extended method was designed for imbalanced data, it can also be used on reasonably balanced data to improve the efficiency of mining the rules needed to provide automatic explanations. Among the existing automatic explanation methods for machine learning prediction results, our method is the only one that can automatically recommend tailored interventions [33,34]. This capability is desired for many medical applications.
2. We present a guideline to set the values of the parameters used in the extended method (see the Discussion section).
3. We completed the first computer coding implementation of the extended method and explained it in this paper.
4. We demonstrate the effectiveness of the extended method in predicting asthma hospital visits in patients with asthma.

#### Review of Our Original Automatic Explanation Method

##### Main Idea

Our automatic explanation method separates explanation and prediction by employing 2 models concurrently, each for a distinct purpose. The first model is used to make predictions and can be any model that takes continuous and categorical features as its inputs. Usually, we adopt the most accurate model as the first model to avoid lowering the performance measures of the model. The second model uses class-based association rules [35,36] mined from historical data to explain the prediction results of the first model rather than to make predictions. Before using a standard association rule mining method like Apriori to mine the rules [36], each continuous feature is first transformed into a categorical feature through automatic discretization [35,37]. Each rule shows a feature pattern associated with a value *w* of the outcome variable in the form of *q_1_* AND *q_2_* AND … AND *q_n_*→*w*. The values of *n* and *w* can change across rules. For binary classification distinguishing poor versus good outcomes, *w* is usually the poor outcome value. Every item *q_i_* (1≤*i*≤*n*) is a feature-value pair (*f*, *u*) showing feature *f* has value *u* or a value within *u*, depending on whether *u* is a value or a range. The rule points out that a patient’s outcome variable is inclined to have value *w* if the patient fulfills *q_1_*, *q_2_*, ..., and *q_n_*. An example rule is as follows:

- The patient had ≥12 ED visits in the past year

AND the patient had ≥21 distinct medications in all asthma medication orders in the past year

→the patient will incur one or more asthma hospital visits in the subsequent year.

##### The Association Rule Mining and Pruning Processes

The association rule mining process is controlled by 2 parameters: the minimum support threshold *s_min_* and the minimum confidence threshold *c_min_* [36]. For any rule *l*: *q_1_* AND *q_2_* AND … AND *q_n_*→*w*, the percentage of data instances satisfying *q_1_*, *q_2_*, ..., and *q_n_* and linking to *w* is termed *l*’s support showing *l*’s coverage. Among all data instances satisfying *q_1_*, *q_2_*, ..., and *q_n_*, the percentage of data instances linking to *w* is termed *l*’s confidence reflecting *l*’s precision. Our original automatic explanation method uses rules with support ≥*s_min_* and confidence ≥*c_min_*. For binary classification distinguishing poor versus good outcomes, we usually focus on the rules that have right-hand sides containing the poor outcome value.

Usually, numerous association rules have support and confidence ≥*s_min_* and ≥*c_min_*, respectively. To avoid overwhelming the users of the automatic explanation function with too many rules, we used 4 techniques to reduce the number of rules in the second model. First, only features adopted by the first model are used to form rules. Second, a clinician in the automatic explanation function’s design team checks all possible values and value ranges of these features and marks those that could possibly have a positive correlation with the values of the outcome variable reflecting poor outcomes. Only those marked values and value ranges of these features are allowed to show up in the rules. Third, the rules are limited to having no more than a given small number of items on their left-hand sides, as long rules are hard to understand. A typical value of this number is 4. Fourth, each more specific rule is dropped when there exists a more general rule with confidence that is not lower by more than a given threshold τ≥0. More specifically, consider 2 rules, *l_1_* and *l_2_*, whose right-hand sides have the same value. The items on the left-hand side of *l_2_* are a superset of those on the left-hand side of *l_1_*. We drop *l_2_* if *l_1_*’s confidence is ≥*l_2_*’s confidence-τ.

For the association rules remaining after the rule-pruning process, a clinician in the automatic explanation function’s design team gathers zero or more interventions targeting the reason the rule presents. A rule is called actionable if one or more interventions are compiled for it. Usually, each intervention links to one of the feature-value pair items on the rule’s left-hand side. Such an item is called actionable. Thus, an actionable rule contains at least 1 actionable item. To expedite the intervention compilation process, the clinician can identify all of the actionable items and compile interventions for each of them. All of the interventions linking to the actionable items on a rule’s left-hand side are automatically connected to the rule.

Our automatic explanation method uses 2 types of knowledge manually compiled by a clinician: the values and value ranges of the features that could possibly have a positive correlation with the outcome variable’s values reflecting poor outcomes and the interventions for the actionable items. Our automatic explanation method is fully automatic, except for the knowledge compilation step.

##### The Explanation Method

For each patient for whom the first model predicts a poor outcome, we explain the prediction result by listing the association rules in the second model whose right-hand sides have the corresponding poor outcome value and whose left-hand sides are fulfilled by the patient, whereas ignoring the rules in the second model whose right-hand sides have a value that differs from the corresponding poor outcome value and whose left-hand sides are fulfilled by the patient. Every rule listed offers a reason why the patient is predicted to have a poor outcome. For each actionable rule listed, the linked interventions are displayed next to it. This helps the user of the automatic explanation function find tailored inventions suitable for the patient. Typically, the rules in the second model describe common reasons for poor outcomes. However, some patients will have poor outcomes for rare reasons not covered by these rules. Consequently, the second model can provide explanations for most, but not all, of the patients for whom the first model predicts poor outcomes.

#### The Previously Outlined Extension of the Original Automatic Explanation Method

Our original automatic explanation method was designed for reasonably balanced data and is unsuitable for imbalanced data, where one value of the outcome variable appears much less often than another. If the minimum support threshold *s_min_* is large on imbalanced data, we cannot obtain enough association rules for the outcome variable's rare values. Consequently, for a large portion of the first model's prediction results on these values, we cannot give any explanation. Conversely, if *s_min_* is too small, the rule mining process will generate too many rules as intermediate results, most of which will be filtered out in the end. This easily exhausts computer memory and makes the rule mining process extremely slow. In addition, many overfitted rules will be produced in the end, making it difficult for clinicians to examine the mined rules.

In our recently outlined extension of the original automatic explanation method [31,32] to handle imbalanced data, we replace support with value-specific support termed commonality [38]. For any rule *l*: *q_1_* AND *q_2_* AND ... AND *q_n_*→*w*, among all data instances linking to *w*, the percentage of data instances satisfying *q_1_*, *q_2_*, ..., and *q_n_* is termed *l*’s commonality showing *l*’s coverage within the context of *w*. Moreover, we replace the minimum support threshold *s_min_* with the minimum commonality threshold *m_min_*. Instead of using rules whose support is ≥*s_min_* and whose confidence is ≥ the minimum confidence threshold *c_min_*, we used rules whose commonality is ≥*m_min_* and whose confidence is ≥*c_min_*.

Each value of the outcome variable falls into one of 2 possible cases. In the first case, the value is interesting and represents an abnormal case. The prediction results of this value require attention and explanations. In the second case, the value is uninteresting and represents a normal case. The prediction results of this value require neither special attention nor explanation. Typically, each interesting value is a rare one reflecting poor outcomes. The second model contains only the association rules related to interesting values. To mine these rules, we proceeded in 2 steps:

- Step 1: For each interesting value *w*, we applied a standard association rule mining method like Apriori [36] to the set *S_w_* of data instances linking to *w* to mine the rules related to *w* and with support on *S_w_* ≥ the minimum commonality threshold *m_min_*. These rules have commonality ≥*m_min_* on the set *S_all_* of all data instances. As *S_w_* is much smaller than *S_all_*, mining these rules from *S_w_* is much more efficient than first applying the association rule mining method to *S_all_* to obtain the rules with support on *S_all_* ≥*m_min_*×|*S_w_*|/|*S_all_*|, and then filtering out those rules unrelated to *w*. Here, |*S*| denotes the cardinality of set *S*.
- Step 2: For each rule mined from *S_w_*, we compute its confidence on *S_all_*. We keep it only if its confidence on *S_all_* is ≥ the minimum confidence threshold *c_min_*.

#### Techniques for Efficiently Mining the Association Rules Whose Commonality is ≥*m_min_*, Computing Their Confidence, and Eliminating Those Rules Whose Confidence is <*c_min_* in the Extended Automatic Explanation Method

When the set *S_all_* of all data instances includes many data instances and features, we often find that the set *S_w_* of data instances linking to an interesting value *w* contains many data instances, and the first model adopts many features. Without limiting the number of data instances in *S_w_* and the number of features, numerous (eg, several billion) association rules would be mined from *S_w_* in Step 1. This makes the computer easily run out of memory and the rule mining process extremely slow. In addition, many rules will be produced at the end, making it difficult for clinicians to examine them. To address this issue, we can use one or more of the following approaches:

1. We take a random sample of data instances *S_sample_* from *S_all_* and use *S_sample_* rather than *S_all_* to mine the rules [39].
2. Before the rule mining process starts, each data instance is transformed into a transaction. To reduce its size, we remove from the transaction those values and value ranges that the clinician in the automatic explanation function’s design team marks as not allowed to show up in any of the rules.
3. Instead of using all of the features adopted by the first model, we use only the top features to mine the rules. Usually, the top features contain most of the predictive power possessed by all features adopted by the first model [23]. If the machine learning algorithm used to build the first model is like XGBoost [24] or random forest, which automatically computes each feature’s importance value, the top features are those with the highest importance values. Otherwise, if the machine learning algorithm used to build the first model does not automatically compute each feature’s importance value, we can use an automatic feature selection method [40] such as the information gain method to choose the top features. Alternatively, we can use XGBoost or random forest to construct a model, automatically compute each feature’s importance value, and choose the top features with the highest importance values.

In the following, we focus on the case of using the set *S_all_* of all data instances to mine the association rules. The case of using a random sample of data instances *S_sample_* from *S_all_* to mine the rules can be handled in a similar way. To compute the rules’ confidence values, we transformed *S_all_* to the matrix format, with each row of the matrix linking to a distinct data instance and each column of the matrix linking to a distinct value or value range of a feature. For medical data, the matrix is often not very sparse. In this case, we can use a separate bitmap to represent each column of the matrix in a condensed manner. For each rule *l*: *q_1_* AND *q_2_* AND ... AND *q_n_*→*w*, we performed efficient bitmap operations to pinpoint the data instances satisfying *q_1_*, *q_2_*, ..., and *q_n_* and needed for computing *l*’s confidence.

Among all the mined association rules related to an interesting value *w*, we needed to identify those whose confidence on the set *S_all_* of all data instances is ≥ the minimum confidence threshold *c_min_*. To expedite the identification process, we proceeded as follows: for each rule *l*: *q_1_* AND *q_2_* AND ... AND *q_n_*→*w*, let *l_w_* denote the number of data instances satisfying *q_1_*, *q_2_*, ..., and *q_n_* and linking to *w*, and *l_¬w_* denote the number of data instances satisfying *q_1_*, *q_2_*, ..., and *q_n_* and not linking to *w*. Our key insight was that *l*’s confidence on *S_all_*
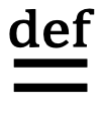
 *l_w_*/(*l_w_*+*l_¬w_*) is <*c_min_* if and only if *l_¬w_* is >*T_l_*
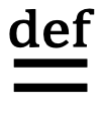
 *l_w_*×(1-*c_min_*)/*c_min_*. We partitioned *S_all_* into 2 subsets: *S_w_* containing all of the data instances linking to *w* and *S_¬w_* containing all of the data instances not linking to *w*. Using the bitmap method mentioned above, we went over all of the data instances in *S_w_* to compute *l_w_*. Then, we went over the data instances in *S_¬w_* one by one to count the data instances satisfying *q_1_*, *q_2_*, ..., and *q_n_* and not linking to *w*. Once this count is >*T_l_*, we know *l*’s confidence on *S_all_* is <*c_min_*, stop the counting process, and drop *l*. This saves the overhead of going through the remaining data instances in *S_¬w_* to compute *l_¬w_*. Otherwise, if this count is ≤*T_l_* when we reach the last data instance in *S_¬w_*, we keep *l*, obtain *l_¬w_*, and compute *l*’s confidence on *S_all_*, which must be ≥*c_min_*.

#### Computer Coding Implementation

We implemented our extended automatic explanation method in computer code, using a hybrid of the C and R programming languages. As R is an interpreted language and inefficient at handling certain operations on large data sets, we wrote several parts of our code in C to improve our code’s execution speed. Considering that our asthma outcome variable is hard to predict, we limited the association rules to have at most 5 items on their left-hand sides (see the guideline in the *Discussion* section). We set the minimum confidence threshold *c_min_* to 50% and the minimum commonality threshold *m_min_* to 0.2%.

### Data Analysis

#### The Training and Test Set Partitioning

As outcomes came from the subsequent year, our data set included 13 years of effective data (2005-2017) during the 14 years between 2005 and 2018. To mirror the practical use of our XGBoost model and our extended automatic explanation method, the 2005 to 2016 data were used as the training set to train our XGBoost model and mine the association rules used by our extended method. The 2017 data were used as the test set to evaluate the performance of our XGBoost model and extended method. We used the full set of 142 features to make predictions and the top 50 features that our XGBoost model [23] ranked with the highest importance values to mine the association rules. Our XGBoost model reached an AUC of 0.859 using the full set of 142 features [23] and an AUC of 0.857 using the top 50 features.

#### Presenting 5 Example Association Rules Used in the Second Model

To give the reader a concrete feeling of the association rules used in the second model, we randomly chose 5 example rules to present in this paper.

#### Performance Metrics

We evaluated the performance of our extended automatic explanation method in several ways. The main performance metric that we used to show our extended method’s explanation capability was the percentage of patients for whom our extended method could provide explanations among the patients with asthma whom our XGBoost model correctly predicted to incur asthma hospital visits in the subsequent year. We reported both the average number of rules and the average number of actionable rules fitting such a patient. A rule fits a patient if the patient fulfills all of the items on its left-hand side.

As shown in our previous study [27], multiple rules fitting a patient frequently differ from each other by a single feature-value pair item on their left-hand sides. When many rules fit a patient, the amount of nonredundant information embedded in them is often much less than the number of these rules. To give a full picture of the information richness of the automatic explanations provided for the patients, we present 3 distributions of the patients with asthma whom our XGBoost model correctly predicted to incur asthma hospital visits in the subsequent year: (1) by the number of rules fitting a patient, (2) by the number of actionable rules fitting a patient, and (3) by the number of distinct actionable items appearing in all of the rules fitting a patient.

## Results

### Our Patient Cohort’s Demographic and Clinical Characteristics

Every data instance targets a distinct pair of a patient with asthma and a year. Table 1 lists the demographic and clinical characteristics of our patient cohort between 2005 and 2016, which included 182,245 patients. Table 2 lists the demographic and clinical characteristics of our patient cohort in 2017, which included 19,256 patients. These 2 sets of characteristics are reasonably similar. Between 2005 and 2016, 3.59% (11,332/315,308) of data instances were related to asthma hospital visits in the subsequent year. In 2017, this percentage was 4.22% (812/19,256).

**Table 1:** Demographic and clinical characteristics of the Intermountain Healthcare patients with asthma between 2005 and 2016.

Characteristics			Data instances related to no asthma hospital visit in the subsequent year (n=303,976), n (%)		Data instances related to asthma hospital visits in the subsequent year (n=11,332), n (%)		Data instances (n=315,308), n (%)
**Gender**							
	Female	181,928 (59.85)		6163 (54.39)		188,091 (59.65)	
	Male	122,048 (40.15)		5169 (45.61)		127,217 (40.35)	
**Age (years)**							
	≥65	46,260 (15.22)		621 (5.48)		46,881 (14.87)	
	18 to 65	172,436 (56.73)		5003 (44.15)		177,439 (56.27)	
	6 to <18	50,572 (16.64)		2590 (22.86)		53,162 (16.86)	
	<6	34,708 (11.42)		3118 (27.52)		37,826 (12.00)	
**Ethnicity**							
	Non-Hispanic	244,442 (80.41)		8157 (71.98)		252,599 (80.11)	
	Hispanic	27,014 (8.89)		2279 (20.11)		29,293 (9.29)	
	Unknown or not reported	32,520 (10.70)		896 (7.91)		33,416 (10.60)	
**Race**							
	White	273,206 (89.88)		9420 (83.13)		282,626 (89.63)	
	Native Hawaiian or other Pacific Islander	3877 (1.28)		411 (3.63)		4288 (1.36)	
	Black or African American	5291 (1.74)		460 (4.06)		5751 (1.82)	
	Asian	2120 (0.70)		77 (0.68)		2197 (0.70)	
	American Indian or Alaska Native	2295 (0.76)		214 (1.89)		2509 (0.80)	
	Unknown or not reported	17,187 (5.65)		750 (6.62)		17,937 (5.69)	
**Duration of asthma (years)**							
	>3	76,810 (25.27)		3666 (32.35)		80,476 (25.52)	
	≤3	227,166 (74.73)		7666 (67.65)		234,832 (74.48)	
**Insurance**							
	Self-paid or charity	26,611 (8.75)		1902 (16.78)		28,513 (9.04)	
	Public	76,916 (25.30)		3238 (28.57)		80,154 (25.42)	
	Private	200,449 (65.94)		6192 (54.64)		206,641 (65.54)	
**Smoking status**							
	Never smoker or unknown	251,501 (82.74)		8952 (79.00)		260,453 (82.60)	
	Former smoker	18,735 (6.16)		569 (5.02)		19,304 (6.12)	
	Current smoker	33,740 (11.10)		1811 (15.98)		35,551 (11.28)	
**Comorbidity**							
	Sleep apnea	20,421 (6.72)		471 (4.16)		20,892 (6.63)	
	Sinusitis	14,164 (4.66)		592 (5.22)		14,756 (4.68)	
	Premature birth	5102 (1.68)		440 (3.88)		5542 (1.76)	
	Obesity	35,215 (11.58)		1076 (9.50)		36,291 (11.51)	
	Gastroesophageal reflux	54,887 (18.06)		1309 (11.55)		56,196 (17.82)	
	Eczema	4484 (1.48)		443 (3.91)		4927 (1.56)	
	Cystic fibrosis	447 (0.15)		11 (0.10)		458 (0.15)	
	Chronic obstructive pulmonary disease	12,496 (4.11)		391 (3.45)		12,887 (4.09)	
	Bronchopulmonary dysplasia	394 (0.13)		35 (0.31)		429 (0.14)	
	Anxiety or depression	55,245 (18.17)		1716 (15.14)		56,961 (18.07)	
	Allergic rhinitis	4534 (1.49)		181 (1.60)		4715 (1.50)	
**Asthma medication prescription**							
	Systemic corticosteroid	129,318 (42.54)		7324 (64.63)		136,642 (43.34)	
	Short-acting, inhaled beta-2 agonist	121,983 (40.13)		7545 (66.58)		129,528 (41.08)	
	Mast cell stabilizer	114 (0.04)		7 (0.06)		121 (0.04)	
	Long-acting beta-2 agonist	1744 (0.57)		69 (0.61)		1813 (0.58)	
	Leukotriene modifier	33,187 (10.92)		2320 (20.47)		35,507 (11.26)	
	Inhaled corticosteroid/long-acting beta-2 agonist combination	42,796 (14.08)		2196 (19.38)		44,992 (14.27)	
	Inhaled corticosteroid	73,566 (24.20)		4539 (40.05)		78,105 (24.77)	

**Table 2:** Demographic and clinical characteristics of the Intermountain Healthcare patients with asthma in 2017.

Characteristics		Data instances related to no asthma hospital visit in the subsequent year (n=18,444), n (%)	Data instances related to asthma hospital visits in the subsequent year (n=812), n (%)	Data instances (n=19,256), n (%)
**Gender**				
	Female	11,001 (59.65)	439 (54.06)	11,440 (59.41)
	Male	7443 (40.35)	373 (45.94)	7816 (40.59)
**Age (years)**				
	≥65	3833 (20.78)	46 (5.67)	3879 (20.14)
	18 to 65	9879 (53.56)	386 (47.54)	10,265 (53.31)
	6 to <18	3054 (16.56)	181 (22.29)	3235 (16.80)
	<6	1678 (9.10)	199 (24.51)	1877 (9.75)
**Ethnicity**				
	Non-Hispanic	16,242 (88.06)	618 (76.11)	16,860 (87.56)
	Hispanic	2020 (10.95)	192 (23.65)	2212 (11.49)
	Unknown or not reported	182 (0.99)	2 (0.25)	184 (0.96)
**Race**				
	White	17,025 (92.31)	681 (83.87)	17,706 (91.95)
	Native Hawaiian or other Pacific Islander	299 (1.62)	47 (5.79)	346 (1.80)
	Black or African American	361 (1.96)	42 (5.17)	403 (2.09)
	Asian	195 (1.06)	10 (1.23)	205 (1.06)
	American Indian or Alaska Native	146 (0.79)	13 (1.60)	159 (0.83)
	Unknown or not reported	418 (2.27)	19 (2.34)	437 (2.27)
**Duration of asthma (years)**				
	>3	7734 (41.93)	389 (47.91)	8123 (42.18)
	≤3	10,710 (58.07)	423 (52.09)	11,133 (57.82)
**Insurance**				
	Self-paid or charity	1136 (6.16)	142 (17.49)	1278 (6.64)
	Public	4920 (26.68)	208 (25.62)	5128 (26.63)
	Private	12,388 (67.17)	462 (56.90)	12,850 (66.73)
**Smoking status**				
	Never smoker or unknown	13,956 (75.67)	583 (71.80)	14,539 (75.50)
	Former smoker	2243 (12.16)	83 (10.22)	2326 (12.08)
	Current smoker	2245 (12.17)	146 (17.98)	2391 (12.42)
**Comorbidity**				
	Sleep apnea	2925 (15.86)	78 (9.61)	3003 (15.60)
	Sinusitis	746 (4.04)	34 (4.19)	780 (4.05)
	Premature birth	435 (2.36)	41 (5.05)	476 (2.47)
	Obesity	3389 (18.37)	116 (14.29)	3505 (18.20)
	Gastroesophageal reflux	3477 (18.85)	71 (8.74)	3548 (18.43)
	Eczema	273 (1.48)	34 (4.19)	307 (1.59)
	Cystic fibrosis	94 (0.51)	1 (0.12)	95 (0.49)
	Chronic obstructive pulmonary disease	1033 (5.60)	23 (2.83)	1056 (5.48)
	Bronchopulmonary dysplasia	12 (0.07)	3 (0.37)	15 (0.08)
	Anxiety or depression	3815 (20.68)	131 (16.13)	3946 (20.49)
	Allergic rhinitis	382 (2.07)	10 (1.23)	392 (2.04)
**Asthma medication prescription**				
	Systemic corticosteroid	11,327 (61.41)	693 (85.34)	12,020 (62.42)
	Short-acting, inhaled beta-2 agonist	13,046 (70.73)	739 (91.01)	13,785 (71.59)
	Mast cell stabilizer	8 (0.04)	0 (0.00)	8 (0.04)
	Long-acting beta-2 agonist	47 (0.25)	5 (0.62)	52 (0.27)
	Leukotriene modifier	3364 (18.24)	209 (25.74)	3573 (18.56)
	Inhaled corticosteroid/long-acting beta-2 agonist combination	4178 (22.65)	222 (27.34)	4400 (22.85)
	Inhaled corticosteroid	6817 (36.96)	424 (52.22)	7241 (37.60)

For each demographic or clinical characteristic, Table 3 presents the statistical test results on whether the data instances related to asthma hospital visits in the subsequent year and those related to no asthma hospital visit in the subsequent year had the same distribution. When the *P* value was ≥.05, the 2 sets of data instances had the same distribution. Otherwise, they had different distributions. All *P* values <.05 are shown in italics in Table 3.

**Table 3:** For each demographic or clinical characteristic, the statistical test results on whether the data instances related to asthma hospital visits in the subsequent year and those related to no asthma hospital visit in the subsequent year had the same distribution.

Characteristics		*P* value for the 2005-2016 data	*P* value for the 2017 data
Gender		*<.001* ^a,^ ^b^	*.002* ^a^
Age (years)		*<.001* ^c^	*<.001* ^c^
Ethnicity		*<.001* ^a^	*<.001* ^a^
Race		*<.001* ^a^	*<.001* ^a^
Duration of asthma (years)		*<.001* ^c^	*<.001* ^c^
Insurance category		*<.001* ^a^	*<.001* ^a^
Smoking status		*<.001* ^a^	*<.001* ^a^
**Comorbidity**			
	Sleep apnea	*<.001* ^a^	*<.001* ^a^
	Sinusitis	*.006* ^a^	.91^a^
	Premature birth	*<.001* ^a^	*<.001* ^a^
	Obesity	*<.001* ^a^	*.004* ^a^
	Gastroesophageal reflux	*<.001* ^a^	*<.001* ^a^
	Eczema	*<.001* ^a^	*<.001* ^a^
	Cystic fibrosis	.21^a^	.20^a^
	Chronic obstructive pulmonary disease	*<.001* ^a^	*<.001* ^a^
	Bronchopulmonary dysplasia	*<.001* ^a^	*.02* ^a^
	Anxiety or depression	*<.001* ^a^	*.002* ^a^
	Allergic rhinitis	.38^a^	.13^a^
**Asthma medication prescription**			
	Systemic corticosteroid	*<.001* ^a^	*<.001* ^a^
	Short-acting, inhaled beta-2 agonist	*<.001* ^a^	*<.001* ^a^
	Mast cell stabilizer	.29^a^	>.99^a^
	Long-acting beta-2 agonist	.67^a^	.11^a^
	Leukotriene modifier	*<.001* ^a^	*<.001* ^a^
	Inhaled corticosteroid/long-acting beta-2 agonist combination	*<.001* ^a^	*.002* ^a^
	Inhaled corticosteroid	*<.001* ^a^	*<.001* ^a^

^a^*P* values obtained by performing the chi-square two-sample test.

^b^*P* values <.05 marked in italics.

^c^*P* values obtained by performing the Cochran-Armitage trend test [41].

### The Number of Association Rules Left at Different Phases of Rule Mining and Pruning Processes

**Figure 1:**
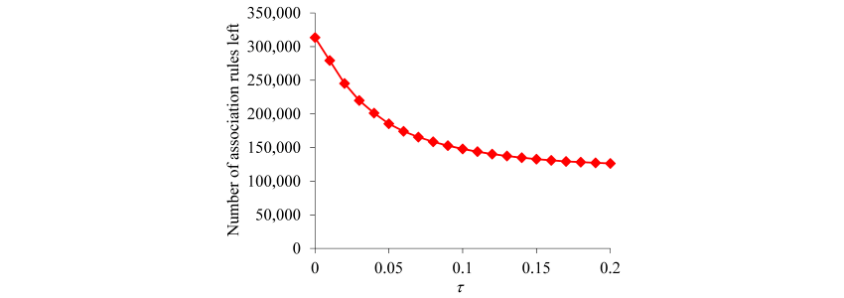
The number of association rules left versus τ.

The association rules used in the second model were mined on the training set. Using the top 50 features that were ranked by our XGBoost model with the highest importance values, we obtained 559,834 association rules. Figure 1 presents the number of rules left versus the confidence difference threshold τ. Recall that each more specific rule is dropped when there exists a more general rule whose confidence is not lower by more than τ. Initially, when τ is small, the number of rules left decreases quickly as τ increases. Once τ becomes 0.15 or larger, the number of rules left approaches an asymptote. Accordingly, in our computer coding implementation, we set τ to 0.15, resulting in 132,816 remaining rules.

A clinical expert on asthma (MJ) in our team marked the values and value ranges of the top 50 features that could possibly have a positive correlation with future asthma hospital visits. After dropping the rules including any other value or value range, 124,506 rules were left. Each rule explains why a patient is predicted to incur one or more asthma hospital visits in the subsequent year. Almost all (124,502/124,506, 100.00%) of these rules were actionable. The left-hand sides of these rules contain various combinations of 208 distinct items related to 50 features.

### Example Association Rules in the Second Model

Table 4 presents 5 sample association rules randomly chosen from the 124,502 actionable rules used in the second model.

**Table 4:** Five sample association rules.

Item on the left-hand side of the rule		Implication of the item	Intervention compiled for the item
**Rule 1: The patient had ≥12 ED^a^ visits in the past year AND the patient had ≥21 distinct medications in all of the asthma medication orders in the past year → the patient will incur one or more asthma hospital visits in the subsequent year.**			
	The patient had ≥12 ED visits in the past year	Having many ED visits reflects poor asthma control	Implement control strategies to avoid the need for emergency care
	The patient had ≥21 distinct medications in all of the asthma medication orders in the past year	Using many asthma medications reflects poor asthma control	Tailor prescribed asthma medications and help the patient maximize asthma control medication adherence
**Rule 2: The patient had ≥9 distinct asthma medication prescribers in the past year AND the block group where the patient lives has a national health literacy score [42] ≤244 AND the patient had ≥21 distinct medications in all of the asthma medication orders in the past year → the patient will incur one or more asthma hospital visits in the subsequent year.**			
	The patient had ≥9 distinct asthma medication prescribers in the past year	Having many asthma medication prescribers reflects poor care continuity, which often leads to poor outcomes	Provide the patient with social resources to address social chaos that leads to ineffective access to health care
	The block group where the patient lives has a national health literacy score ≤244	Having low health literacy is correlated with poor outcomes	Improve education access in the area where the patient lives to help increase health literacy
**Rule 3: The patient had a total of ≥25 units of systemic corticosteroids ordered in the past year AND the patient had ≥12 ED visits in the past year AND the patient is Hispanic → the patient will incur one or more asthma hospital visits in the subsequent year.**			
	The patient had a total of ≥25 units of systemic corticosteroids ordered in the past year	Systemic corticosteroids are one type of asthma medication intended for short-term use to relieve acute asthma exacerbations. Using a lot of systemic corticosteroids reflects poor asthma control	Tailor prescribed asthma medications and help the patient maximize asthma control medication adherence
	The patient is Hispanic	In the US, Hispanic people have a disproportionately high rate of poor asthma outcomes	—^b^
**Rule 4: The patient had ≥4 major visits for asthma in the past year AND the patient is between 11 and 35 years old AND the patient had no outpatient visit in the past year AND the average length of an inpatient stay of the patient in the past year is >1.75 and ≤2.95 days → the patient will incur one or more asthma hospital visits in the subsequent year.**			
	The patient had ≥4 major visits for asthma in the past year	As defined in our paper [23], a major visit for asthma is an inpatient stay or ED visit having an asthma diagnosis code, or an outpatient visit having a primary diagnosis of asthma. Intuitively, all else being equal, a patient having major visits for asthma has a higher likelihood of incurring future asthma hospital visits than a patient having only outpatient visits with asthma as a secondary diagnosis	Implement control strategies to avoid the need for emergency care
	The average length of an inpatient stay of the patient in the past year is >1.75 and ≤2.95 days	Having inpatient stays reflects poor asthma control	Implement control strategies to avoid the need for emergency care
	The patient had no outpatient visit in the past year	For good asthma management, a patient with asthma is supposed to see the primary care provider regularly. Having no outpatient visit often implies that the patient has no primary care provider	Help the patient obtain a primary care provider if the patient does not already have one
**Rule 5: The patient had ≥4 major visits for asthma in the past year AND the patient's last ED visit is within the last 49 days AND the patient had between 6 and 8 distinct asthma medication prescribers in the past year AND the patient had a total of ≥36 units of asthma medications ordered in the past year AND >23.7% and ≤33.3% of families in the block group where the patient lives are below 150% of the federal poverty level → the patient will incur one or more asthma hospital visits in the subsequent year.**			
	The patient’s last ED visit is within the last 49 days	Having a recent ED visit reflects poor asthma control	Implement control strategies to avoid the need for emergency care
	The patient had a total of ≥36 units of asthma medications ordered in the past year	Taking many asthma medications reflects poor asthma control	Tailor prescribed asthma medications and help the patient maximize asthma control medication adherence
	>23.7% and ≤33.3% of families in the block group where the patient lives are below 150% of the federal poverty level	Poverty correlates with poor outcomes	Provide living wage programs in the area where the patient lives to increase resources for health care

^a^ED: emergency department.

^b^Not applicable.

### Performance Measures Reached by the Extended Automatic Explanation Method

Our extended automatic explanation method was assessed on the test set. This method explained the prediction results for 92.4% (182/197) of the adults with asthma (age ≥18 years) and 87.5% (209/239) of the children with asthma (age <18 years) for whom our XGBoost model correctly predicted the occurrence of asthma hospital visits in the subsequent year. Combined, our extended method explained the prediction results for 89.7% (391/436) of the patients with asthma whom our XGBoost model correctly predicted to incur asthma hospital visits in the subsequent year. For each such patient, our extended method offered an average of 974.01 (SD 1600.48) explanations, 974.00 (SD 1600.47) of which were actionable. Each explanation came from 1 rule. When confined to using actionable rules, our extended method explained the prediction results for 89.7% (391/436) of the patients with asthma for whom our XGBoost model correctly predicted the occurrence of asthma hospital visits in the subsequent year.

For the patients for whom our extended automatic explanation method could offer explanations of our XGBoost model’s correct prediction results of incurring asthma hospital visits in the subsequent year, the average number of distinct actionable items appearing in all of the rules fitting a patient was 21.50 (SD 8.71). This number is much less than 974.01, the average number of actionable rules fitting such a patient.

**Figure 2:**
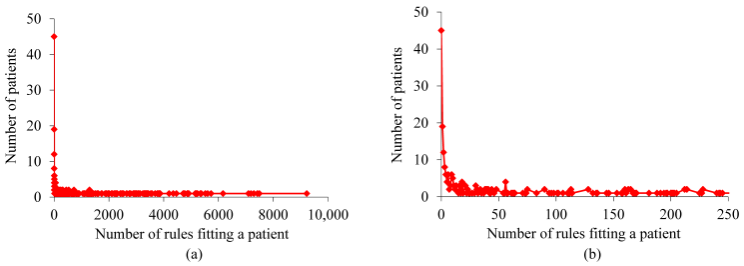
Distribution of patients by the number of rules fitting a patient for the patients with asthma whom our extreme gradient boosting model correctly predicted to incur asthma hospital visits in the subsequent year. (a) When no limit is placed on the number of rules fitting a patient. (b) When the number of rules fitting a patient is ≤250.

For the patients with asthma whom our XGBoost model correctly predicted to incur asthma hospital visits in the subsequent year, Figure 2 shows the distribution of patients by the number of rules fitting a patient. This distribution has a long tail and is highly skewed toward the left. As the number of rules fitting a patient becomes larger, the number of patients to each of whom this number of rules apply is inclined to drop nonmonotonically. The largest number of rules fitting a patient is high, 9223, although only 1 patient fits such a high number of rules.

**Figure 3:**
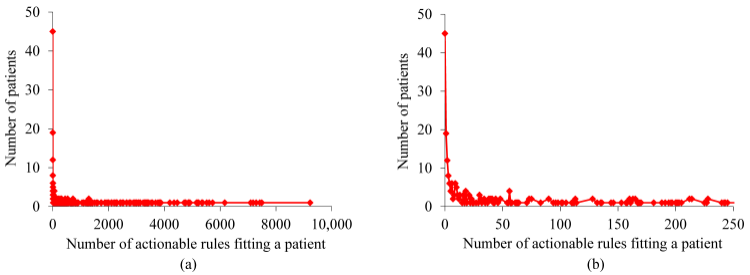
Distribution of patients by the number of actionable rules fitting a patient for the patients with asthma whom our extreme gradient boosting model correctly predicted to incur asthma hospital visits in the subsequent year. (a) When no limit is placed on the number of actionable rules fitting a patient. (b) When the number of actionable rules fitting a patient is ≤250.

For the patients with asthma whom our XGBoost model correctly predicted to incur asthma hospital visits in the subsequent year, Figure 3 shows the distribution of patients by the number of actionable rules fitting a patient. This distribution is similar to that shown in Figure 2. The largest number of actionable rules fitting a patient is high, 9223, although only 1 patient fits such a high number of actionable rules.

**Figure 4:**
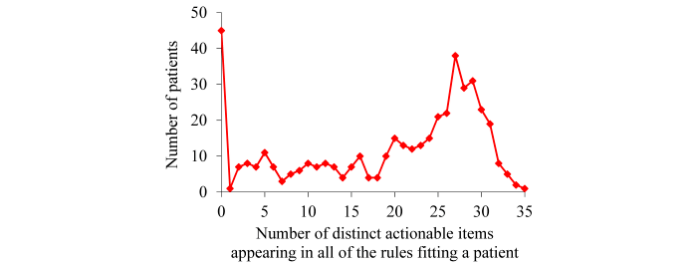
Distribution of patients by the number of distinct actionable items appearing in all of the rules fitting a patient for the patients with asthma whom our extreme gradient boosting model correctly predicted to incur asthma hospital visits in the subsequent year.

For the patients with asthma whom our XGBoost model correctly predicted to incur asthma hospital visits in the subsequent year, Figure 4 exhibits the distribution of patients by the number of distinct actionable items appearing in all of the rules fitting a patient. The largest number of distinct actionable items appearing in all of the rules fitting a patient is 35, much smaller than the largest number of (actionable) rules fitting a patient. Frequently, 2 or more actionable items appearing in the rules fitting a patient link to the same set of interventions. For example, the intervention of tailoring prescribed asthma medications and helping the patient maximize asthma control medication adherence links to several value ranges of multiple medication-related features.

Our extended automatic explanation method could offer explanations for 69.2% (562/812) of patients with asthma who will incur asthma hospital visits in the subsequent year.

To evaluate the generalizability of our extended automatic explanation method for predicting asthma hospital visits, we tested our method on the University of Washington Medicine data and Kaiser Permanente Southern California data. The results we obtained are similar to the abovementioned results and are detailed in 2 separate papers [43,44].

## Discussion

### Principal Findings

We developed a method to automatically offer rule-formed explanations for any machine learning model’s prediction results on imbalanced tabular data without lowering the performance measures of the model. We showed that this method explained the prediction results for 89.7% (391/436) of the patients with asthma whom our XGBoost model correctly predicted to incur asthma hospital visits in the subsequent year. This percentage is high enough for routine clinical use of this method. After further improvement of its accuracy, our asthma outcome prediction model coupled with the automatic explanation function could be used for decision support to guide the allocation of limited asthma care management resources. This could help boost asthma outcomes and reduce resource use and costs.

Our extended automatic explanation method could offer explanations for 69.2% (562/812) of the patients with asthma who will incur asthma hospital visits in the subsequent year. This percentage is smaller than the success rate of 89.7% (391/436) for our extended automatic explanation method to explain the correct prediction results of our XGBoost model of incurring asthma hospital visits in the subsequent year. One possible reason is that the prediction results of the association rules are correlated with the prediction results of our XGBoost model. Among the patients with asthma who will incur asthma hospital visits in the subsequent year and on whom our XGBoost model gave incorrect predictions, many are difficult cases for any model to correctly predict or explain their outcomes. Among the patients with asthma whom our XGBoost model correctly predicted to incur asthma hospital visits in the subsequent year, many are easy cases for using association rules to explain the outcomes of these cases.

Asthma in adults differs from asthma in children. As shown in a previous study [23], the AUC of our XGBoost model for adults with asthma was 0.034 higher than that for children with asthma, that is, the outcome is easier to predict for adults with asthma than for children with asthma. Intuitively, the degree of difficulty in predicting the outcome is positively correlated with that of using association rules to explain the prediction results of the model, as each rule is a small predictive model. Hence, our extended automatic explanation method explained the prediction results for a larger portion of the adults with asthma than the children with asthma for whom our XGBoost model correctly predicted the occurrence of asthma hospital visits in the subsequent year.

### A Guideline for Setting the Values of the Parameters Used in Our Extended Automatic Explanation Method

Our extended automatic explanation method has 4 parameters: the maximum number of items *l_max_* allowed on the left-hand side of an association rule, the minimum commonality threshold *m_min_*, the minimum confidence threshold *c_min_*, and the confidence difference threshold τ. These parameters significantly affect the performance of the method. Our previous papers [31,32] outlined the method but gave no guideline for setting the values of these parameters. We offer such a guideline here.

The maximum number of items *l_max_* allowed on the left-hand side of an association rule is usually small, as long rules are difficult to understand [35]. Our previous study [27] showed that for an outcome variable that is relatively easy to predict, an *l_max_* of 4 works well for automatic explanation. When the outcome variable is hard to predict, we can increase *l_max_* slightly to a number such as 5. Without making the rules too complex to understand, this helps ensure that the second model can provide explanations for a large portion of the data instances that the first model correctly predicts to take one of the interesting values of the outcome variable.

In the original paper [38] that proposed the concept of commonality for class-based association rules, mined rules were used to build a classifier. To maximize the accuracy of the classifier, the minimum commonality threshold *m_min_* was set to 14%. However, this value is too high for automatic explanation. With such a high value, we cannot obtain enough rules for the outcome variable’s rare values. Consequently, for a large portion of the first model’s prediction results on these values, we cannot give any explanation. In addition, the mined rules tend to be too general and have low confidence, causing the users of the automatic explanation function to have little trust in the automatically generated explanations. To avoid these problems, for automatic explanation, we recommend setting *m_min_* to a value much smaller than 14%. More specifically, our paper [27] showed that on reasonably balanced data, a minimum support threshold *s_min_* of 1% and a minimum confidence threshold *c_min_* of 50% work well for automatic explanation. By definition, commonality is a value-specific support. Thus, we would expect *m_min_* and *s_min_* to have relatively similar optimal values. Accordingly, we set *m_min_* to a value close to 1% and *c_min_* to a value close to 50%. Although a value close to 50% may not seem so high, it is already much larger than the percentage of data instances linking to an interesting value of the outcome variable. For instance, in our case of predicting asthma hospital visits in patients with asthma, this percentage is 4% [23]. Moreover, a value close to 50% is also much larger than our XGBoost model’s positive predictive value of 22.65%. The concrete values of *m_min_* and *c_min_* depend on the data set and are chosen to meet 2 goals simultaneously and as much as possible. First, the second model can provide explanations for a large portion of the data instances that the first model correctly predicts to take one of the interesting values of the outcome variable. Often, the harder the outcome variable is to predict, the smaller *m_min_* and *c_min_* need to be to meet this goal. Second, *c_min_* is high enough for users of the automatic explanation function to trust the automatically generated explanations.

Recall that during the rule-pruning process, each more specific rule is dropped when there is a more general rule whose confidence is not lower by more than the confidence difference threshold τ. To determine the value of *τ*, we plot the number of rules left versus τ. As our previous paper [27] shows, initially when τ is small, the number of rules left decreases quickly as τ increases. Once τ becomes sufficiently large, the number of rules left approaches an asymptote. This is the place to set the value of τ to strike a balance between cutting the number of rules and retaining high-confidence rules.

### Five Clarifications on Using the Automatic Explanation Function

In practice, our automatic explanation method could produce a paradox. Two patients both fulfilled the left-hand side of the same rule linking to a poor outcome. The first model correctly predicts one of them to have a poor outcome. The automatic explanation function displays the rule to explain this prediction result. Simultaneously, the first model correctly predicts a good outcome on the other patient, for whom the automatic explanation function shows nothing. In this case, one should not think that the automatic explanation function acts incorrectly because it behaves differently in these 2 patients; rather, this difference occurs because the second patient fulfills some items that are not in the rule. These items counter the risk induced by those on the rule’s left-hand side and reduce the second patient’s risk of having a poor outcome to a low level.

When using the automatic explanation function, one needs to remember that the function is intended to serve as a reminder system for decision support rather than a replacement for clinical judgment. The function is used to help the user quickly identify some reasons why a patient is predicted to have a poor outcome and some tailored interventions suitable for the patient. If successful, this helps the clinical user avoid substantial time laboriously reviewing the records of the patient to assess risk factors and devise interventions. This also helps reduce the number of interventions that are suitable for the patient, but the user forgets to consider. In the end, it is still the user who uses his or her own judgment to decide whether to use the prediction result of the first model and apply suggested interventions to the patient. If there is doubt about the appropriateness of the output of the function, the clinical user can always check the records of the patient to resolve the doubt before making the final decisions with the patient.

Different health care systems have different properties and practice patterns. Consequently, the association rules mined from the data of one health care system may or may not directly apply to or work well for another health care system. However, our automatic explanation method is general. It relies on no special property of a specific disease, patient cohort, prediction target, or health care system and can be applied to various predictive modeling problems and health care systems [27,29,30,43,44], regardless of whether the rules mined from the data of 1 health care system generalize to the data of another health care system. For any health care system, we would recommend mining rules from its own data whenever possible, rather than reusing the rules mined from the data of another health care system.

In our test case, the second model contained 124,506 association rules. The left-hand sides of these rules contain various combinations of 208 distinct items related to 50 features. Within 1 day, a clinician in our team (MJ) finished manually compiling the 2 types of knowledge needed by the automatic explanation function: the values and value ranges of the top 50 features that could possibly have a positive correlation with future asthma hospital visits and the interventions for the actionable items. The amount of time needed to perform this manual compilation is moderate and acceptable to the clinicians in our team.

Although many association rules could fit a patient, the total number of distinct items included on their left-hand sides is not large: at most 35. To avoid overwhelming the automatic explanation function’s user, we can use the rule diversification method in our paper [27] to rank these rules. The top few rules are likely to contain nonredundant information and are displayed by default.

### Related Work

As described in a survey paper [33] and a book [34], other researchers previously proposed various methods for automatically explaining the prediction results of machine learning models. These methods often lower the performance measures of the model by replacing the original model with a less accurate model and usually give nonrule-formed explanations. Many of these methods work for only a specific machine learning algorithm rather than for all algorithms. Moreover, none of these methods can automatically recommend tailored interventions. In comparison, our extended automatic explanation method not only offers rule-formed explanations for the prediction results of any machine learning model on tabular data but also recommends tailored interventions without lowering the performance measures of the model [27]. Compared with nonrule-formed explanations, rule-formed explanations are easier to comprehend and can more directly recommend tailored interventions.

Hatwell et al [45] proposed a method to automatically provide rule-formed explanations for the prediction results of an AdaBoost model. This method does not work for non-AdaBoost machine learning algorithms. The rules are unknown before the prediction time and hence cannot be used to automatically recommend tailored interventions at prediction time. In comparison, the rules used in our extended automatic explanation method are precompiled beforehand and used to automatically recommend tailored interventions at prediction time.

### Limitations

This study has 2 limitations that give interesting directions for future work:

1. Our data set contained no information on health care use of the patients outside of Intermountain Healthcare. Consequently, the features were computed using incomplete clinical and administrative data [46-49]. In addition, the prediction target was limited to asthma hospital visits at Intermountain Healthcare rather than asthma hospital visits anywhere. It would be interesting to see how the automatically generated explanations of the prediction results of the model would differ if we have access to more complete clinical and administrative data [50].
2. Our study used 1 predictive modeling problem, predicting asthma hospital visits as the test case. Although our original automatic explanation method [27] has been successfully applied to several predictive modeling problems [29,30], the generalizability of our extended automatic explanation method to other predictive modeling problems beyond predicting asthma hospital visits has not been evaluated. Conducting such evaluations would help inform the utility and refine the implementation of our extended method.

### Conclusions

Using asthma outcome prediction as a demonstration case, this study shows for the first time the feasibility of automatically offering rule-formed explanations for the prediction results of any machine learning model on imbalanced tabular data without lowering the performance measures of the model. After further improvement, our asthma outcome prediction model coupled with the automatic explanation function could be used for decision support to guide the allocation of limited asthma care management resources. This could simultaneously help improve asthma outcomes and reduce resource use and cost.

## Abbreviations

AUC: area under the receiver operating characteristic curve
ED: emergency department
ICD-9: International Classification of Diseases, ninth revision
ICD-10: International Classification of Diseases, tenth revision
XGBoost: extreme gradient boosting
